# Splenic Artery Embolization for the Treatment of Gastric Variceal Bleeding Secondary to Splenic Vein Thrombosis Complicated by Necrotizing Pancreatitis: Report of a Case

**DOI:** 10.1155/2016/1585926

**Published:** 2016-11-07

**Authors:** Hee Joon Kim, Eun Kyu Park, Young Hoe Hur, Yang Seok Koh, Chol Kyoon Cho

**Affiliations:** Department of Surgery, Chonnam National University Medical School, Gwangju, Republic of Korea

## Abstract

Splenic vein thrombosis is a relatively common finding in pancreatitis. Gastric variceal bleeding is a life-threatening complication of splenic vein thrombosis, resulting from increased blood flow to short gastric vein. Traditionally, splenectomy is considered the treatment of choice. However, surgery in necrotizing pancreatitis is dangerous, because of severe inflammation, adhesion, and bleeding tendency. In the Warshaw operation, gastric variceal bleeding is rare, even though splenic vein is resected. Because the splenic artery is also resected, blood flow to short gastric vein is not increased problematically. Herein, we report a case of gastric variceal bleeding secondary to splenic vein thrombosis complicated by necrotizing pancreatitis successfully treated with splenic artery embolization. Splenic artery embolization could be the best treatment option for gastric variceal bleeding when splenectomy is difficult such as in case associated with severe acute pancreatitis or associated with severe adhesion or in patients with high operation risk.

## 1. Introduction

Necrotizing pancreatitis (NP) is present in 10~20% of patients with acute pancreatitis. NP should be managed as conservatively as possible because of a high operative mortality. A step-up approach with less invasive management is widely accepted nowadays for management of NP [1]. Bleeding from gastric varices (GVs) secondary to splenic vein thrombosis (SVT) can be life-threatening. GVs secondary to SVT are caused by the imbalance between splenic inflow and outflow. In the setting of SVT, splenic arterial flow is patent, but splenic vein is occluded. Therefore, SVT leads to the increase in splenic outflow via the short gastric and gastroepiploic veins, known as left-sided or sinistral portal hypertension. The increased pressure within the short gastric vein causes submucosal venous dilatation in gastric fundus. It has a potential risk of massive hemorrhage from gastric varices [2].

Traditionally, a splenectomy is considered as a treatment of choice for bleeding GVs secondary to SVT [3]. However, it is sometimes very difficult and dangerous in the patients with NP because of inflammation, adhesion, and bleeding tendency. In the Warshaw operation—a spleen-preserving distal pancreatectomy with splenic vein resection—the splenic artery and vein are resected with the distal pancreas. The splenic blood flow is preserved through the short gastric vessels and gastroepiploic vessels. In the report about the operative outcome of the Warshaw operation for 23 years, it has been reported that there were no clinical consequences of perigastric varices in 158 patients who underwent the Warshaw operation during a follow-up period of up to 21 years [4]. Although the splenic vein is resected in this procedure, pressure in short gastric and gastroepiploic vein is not increased problematically, because the splenic artery is also resected. No patient who underwent this procedure needed splenectomy due to bleeding GVs. Therefore, the Warshaw operation is considered safe and feasible. This theory can be applicable to treat gastric varices secondary to SVT.

Some authors have reported that splenic artery embolization (SAE) is an effective alternative to splenectomy for the treatment of gastric variceal bleeding with left-sided portal hypertension [2, 5, 6].

Herein, we report a case of bleeding GVs secondary to SVT in NP, successfully managed by SAE without splenectomy.

## 2. Case Report

**Figure 1:**
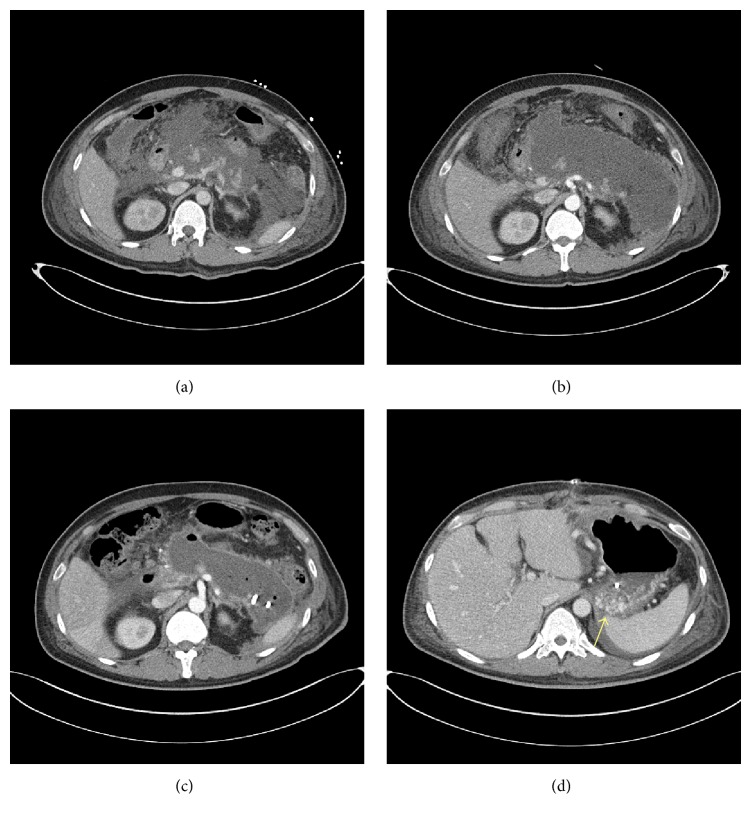
(a) Abdominal CT scan showed severe necrotizing pancreatitis. (b) Abdominal CT scan following 2 weeks of medical treatment. A huge pseudocyst was seen. A percutaneous drainage was performed. (c) Follow-up CT at 4 weeks after external drainage. The function of drain was poor and a huge pseudocyst was still seen. A necrosectomy and surgical drainage was performed. (d) CT scan at 1 week following the operation. Engorged submucosal veins were observed at gastric fundus.

**Figure 2:**
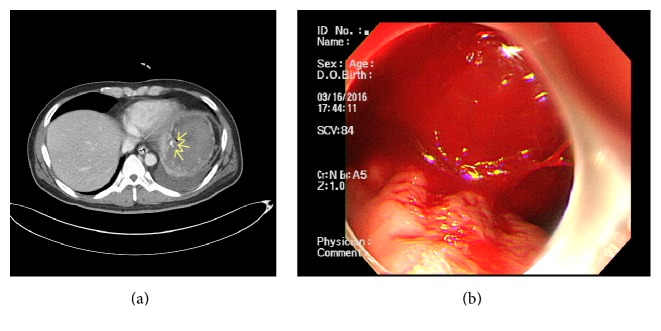
(a) An abdominal CT scan revealed bleeding gastric varices. (b) Esophagogastroduodenoscopy revealing bleeding from fundal gastric varices. Endoscopic hemostasis failed because of large clots and ongoing bleeding.

**Figure 3:**
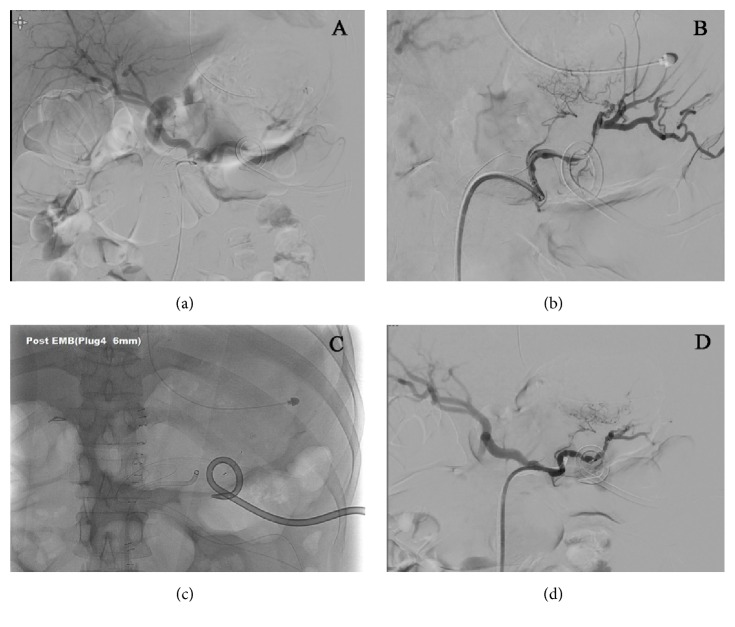
(a and b) Celiac and splenic arteriography showed no extravasation from arterial system. (c) Nonselective splenic artery embolization was performed using vascular plug at distal splenic artery. (d) After embolization, blood flow to spleen was remarkably decreased.

**Figure 4:**
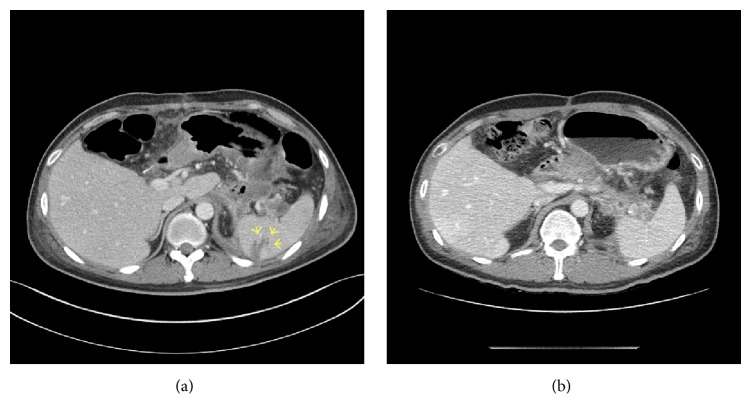
(a) CT scan on 2 days following the SAE. A small area of splenic infarction was seen. (b) CT scan on 4 months following the SAE. The infarcted area was not observed any more.

A 42-year-old man was referred to our hospital for treatment of a necrotizing pancreatitis. He had no history of medication or alcohol consumption. Laboratory studies revealed white blood cell count 20,200/mm^3^, hemoglobin 17.4 g/dL, platelet count 25,8000/mm^3^, serum total bilirubin 2.17 mg/dL, AST 266 U/L, ALT 196 U/L, amylase 1670 U/L, and lipase 2673 U/L. An abdominal CT scan showed acute necrotizing pancreatitis with large amount of peripancreatic necrotic fluid collections (Figure 1(a)). He was admitted to the intensive care unit (ICU) and protease inhibitor and empirical antibiotics treatment was initiated. He presented a cardiac arrest requiring cardiopulmonary resuscitation for 20 minutes, and continuous renal replacement therapy (CRRT) and mechanical ventilation were needed for combined multiorgan failure. An abdominal CT scan following 4 weeks of medical treatment in ICU demonstrated a huge pseudocyst (Figure 1(b)). Despite endoscopic transgastric internal drainage and percutaneous drainage, fever and abdominal pain were not subsided. A follow-up abdominal CT scan after 8 weeks from admission revealed the decreased but still remaining large amount of necrotic collections (Figure 1(c)) and SVT with engorgement of perigastric veins (Figure 1(d)). An emergency operation for necrosectomy and external drainage was performed. On operative field, severe adhesion and bleeding tendency was noted. On postoperative day 13, hematemesis occurred. Blood pressure was 90/60 mmHg and hemoglobin decreased from 11.2 g/dL to 7.6 g/dL. An abdominal CT scan revealed extravasation of contrast media at gastric cardia and fundus (Figure 2(a)). After initial resuscitation, an emergency esophagogastroduodenoscopy (EGD) was performed. It showed a huge clot in the stomach, and active bleeding from gastric fundus was suspected. However, the focus of bleeding could not be identified exactly due to the presence of large clot and ongoing active bleeding (Figure 2(b)). He was referred to intervention unit and underwent an emergency angiography. Celiac and splenic arteriography revealed no active bleeding from arterial system (Figures 3(a)-3(b)). Under suspicion of bleeding GVs, SAE was performed using vascular plug (Figure 3(c)). The vascular plug was placed at distal splenic artery just proximal to branching in splenic hilum. After SAE, splenic flow was remarkably decreased (Figure 3(d)). During the procedure, his systolic blood pressure was 70~80 mmHg and heart rate was 140~150 beats per minute. Four packs of PRCs were transfused after SAE. After the procedure, bleeding stopped immediately, and no more episode of gastrointestinal bleeding was observed. An abdominal CT scan 2 days following the SAE showed no more bleeding. A small splenic infarction was seen, but the infarcted volume was less than 10% of total splenic volume (Figure 4(a)). Any treatment for splenic infarction was not needed. At present, 4 months following the SAE, no more episode of variceal bleeding was observed and no infarcted area of spleen was seen on a follow-up abdominal CT scan (Figure 4(b)).

## 3. Discussion

**Figure 5:**
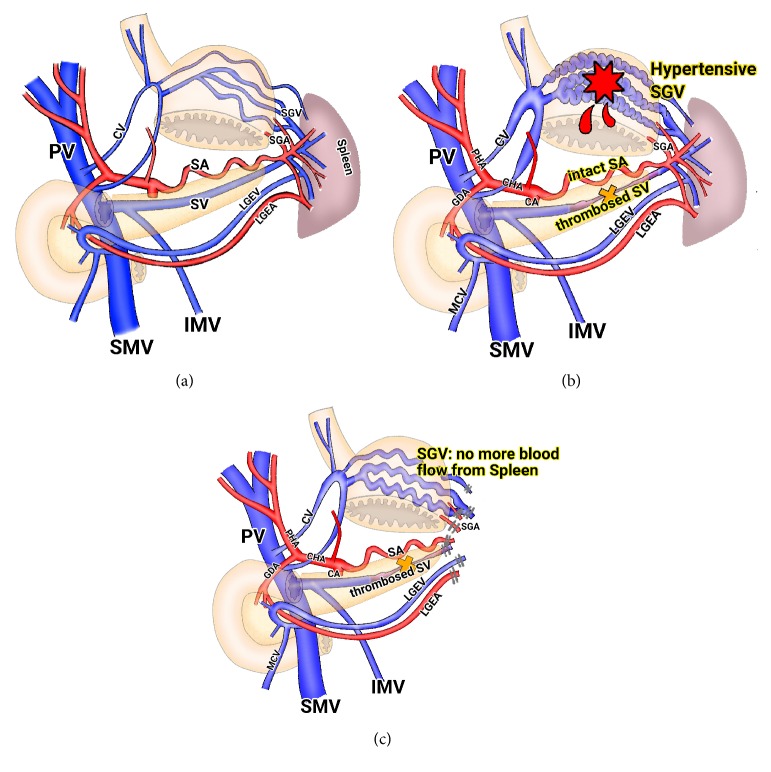
(a) Normal anatomy. The splenic inflows and outflows are balanced. (b) Thrombosed splenic vein and intact splenic artery induces hypertensive short gastric veins. It is a potential source of massive bleeding from gastric varices. (c) The rationale of splenectomy in the setting of gastric variceal bleeding is the complete interruption of blood flow in short gastric veins from spleen. PV, portal vein; SMV, superior mesenteric vein; CV, coronary vein; IMV, inferior mesenteric vein; SA, splenic artery; SV, splenic vein; LGEA, left gastroepiploic artery; LGEV, left gastroepiploic vein; SGA, short gastric artery; SGV, short gastric vein.

It is known that incidence of pancreatitis-induced SVT is 14%, and risk of GI bleeding in these patients is 12% [7]. Thrombosed splenic vein with intact splenic artery induces hypertensive short gastric veins that can develop into varices in the gastric fundal submucosa. These varices are a potential source of significant upper GI bleeding (Figure 5(b)). Traditionally, splenectomy is considered as a treatment of choice for GV bleeding secondary to SVT. The cause of bleeding is the imbalance between inflow and outflow of spleen, not a splenic pathology. Therefore, in this setting, rationale for splenectomy is to interrupt the arterial supply feeding the collateral draining veins and the gastric fundus varices, thus reducing the pressure of the system and consequently the risk of rebleeding [8] (Figure 5(c)). The spleen resection should be forced to interrupt the short gastric vessels, because no more blood outflow is present in spleen after ligation of short gastric veins. Therefore, if we can decrease pressure within short gastric veins and no more bleeding is present, splenectomy is not necessary.

**Figure 6:**
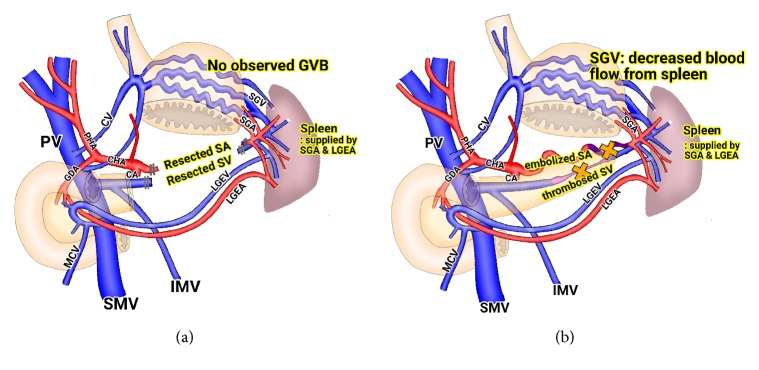
(a) After the Warshaw operation, spleen is supplied by short gastric arteries and left gastroepiploic artery and drained by short gastric veins and left gastroepiploic vein. No gastric variceal bleeding was observed after the operation over 20 years of observation period. (b) After splenic artery embolization, pressure within short gastric veins is decreased. The blood flow of spleen is maintained similar to postoperative circumstance of the Warshaw operation. PV, portal vein; SMV, superior mesenteric vein; CV, coronary vein; IMV, inferior mesenteric vein; SA, splenic artery; SV, splenic vein; LGEA, left gastroepiploic artery; LGEV, left gastroepiploic vein; SGA, short gastric artery; SGV, short gastric vein; GVB, gastric variceal bleeding.

Hematologic and infectious complications can occur after splenectomy [9]. So, many pancreatic surgeons try to preserve spleen during distal pancreatectomy. The Warshaw operation, spleen-preserving distal pancreatectomy (SPDP) with splenic vessels resection, is one of options for distal pancreatectomy. In this procedure, the splenic blood flow is preserved by short gastric vessels and left gastroepiploic vessels. Although the splenic vein is resected in this procedure, pressure in short gastric and gastroepiploic vein is not increased problematically, because the splenic artery is also resected (Figure 6(a)). This procedure has a theoretical risk of splenic infarction and perigastric varices. However, many pancreatic surgeons have reported the safety of the Warshaw operation. Warshaw group have reported that none of 158 patients who underwent the Warshaw operation developed GI bleeding and only 3 (1.9%) patients required a reoperation because of splenic infarction during a follow-up period of up to 21 years [4]. Kim et al. also have reported no clinical significant splenic infarctions or gastric varices in any case after the Warshaw operation [10]. In our center, we performed 19 cases of laparoscopic Warshaw operation from 2014. Splenic infarction was observed in 11 patients; however the infarcted volume was less than 10% in all patients, and no specific treatment was needed. No episode of GI bleeding was observed in all patients.

On the basis of these clinical results after the Warshaw operation, SAE can be a treatment option for GV bleeding induced by SVT. After SAE, blood pressure in short gastric veins is decreased because the blood inflow to spleen is decreased. In addition, spleen can be preserved via short gastric and gastroepiploic blood supply similar to postoperative circumstance of the Warshaw operation (Figure 6(b)). In our case, after SAE, drainage of fresh blood via nasogastric tube was stopped, and hypovolemic shock and anemia are recovered gradually. And very small region of spleen was infarcted, but no specific treatment was needed. Even though gastric and perigastric varices are still observed on abdominal CT scan, no episode of GI bleeding developed during the 4 months of follow-up period.

Any upper abdominal surgery in the patient with severe acute pancreatitis is very dangerous because of sever adhesion, inflammation, obscure anatomy, friable tissue, and bleeding tendency. Especially in necrotizing pancreatitis, a conservative or less invasive treatment is recommended [1]. Therefore, SAE could be the best treatment modality rather than splenectomy in GVs bleeding secondary to SVT in the patients with NP.

Nonselective SAE, embolization of main trunk of splenic artery, seemed more ideal than selective SAE, embolization of each branch of splenic artery, to preserve left gastroepiploic artery. Stone et al. reported 20~30% volume splenic infarction after nonselective SAE [2]. By contrast, Sankararaman et al. planned to embolize 80~90% of the spleen. After selective SAE, huge area of spleen was infarcted [6]. In our case, we performed nonselective SAE; then only small portion of spleen was infarcted.

In conclusion, splenic artery embolization is a safe and feasible modality to manage bleeding gastric varices secondary to splenic vein thrombosis. Especially in necrotizing pancreatitis, it could be the best treatment modality in aspect of “step-up” approach. However, more studies are needed with long-term outcome to clarify the necessity of splenectomy.
